# The Movement Disorder Society Criteria for the Diagnosis of Multiple System Atrophy

**DOI:** 10.1002/mds.29005

**Published:** 2022-04-21

**Authors:** Gregor K. Wenning, Iva Stankovic, Luca Vignatelli, Alessandra Fanciulli, Giovanna Calandra‐Buonaura, Klaus Seppi, Jose‐Alberto Palma, Wassilios G. Meissner, Florian Krismer, Daniela Berg, Pietro Cortelli, Roy Freeman, Glenda Halliday, Günter Höglinger, Anthony Lang, Helen Ling, Irene Litvan, Phillip Low, Yasuo Miki, Jalesh Panicker, Maria Teresa Pellecchia, Niall Quinn, Ryuji Sakakibara, Maria Stamelou, Eduardo Tolosa, Shoji Tsuji, Tom Warner, Werner Poewe, Horacio Kaufmann

**Affiliations:** ^1^ Department of Neurology Innsbruck Medical University Innsbruck Austria; ^2^ Neurology Clinic, University Clinical Center of Serbia, Faculty of Medicine, University of Belgrade Belgrade Serbia; ^3^ IRCCS, Istituto delle Scienze Neurologiche di Bologna Bologna Italy; ^4^ Department of Biomedical and Neuromotor Sciences University of Bologna Bologna Italy; ^5^ Department of Neurology, Dysautonomia Center, Langone Medical Center New York University School of Medicine New York New York USA; ^6^ French Reference Center for MSA, Department of Neurology for Neurodegenerative Diseases University Hospital Bordeaux, 33076 Bordeaux and Institute of Neurodegenerative Diseases, University Bordeaux, CNRS Bordeaux France; ^7^ Department of Medicine University of Otago, Christchurch, and New Zealand Brain Research Institute Christchurch New Zealand; ^8^ Department of Neurodegeneration and Hertie‐Institute for Clinical Brain Research University of Tübingen Tübingen Germany; ^9^ Department of Neurology Christian‐Albrechts‐University Kiel Kiel Germany; ^10^ Department of Neurology, Beth Israel Deaconess Medical Center Harvard Medical School Boston Massachusetts USA; ^11^ Brain and Mind Centre, Faculty of Medicine and Health School of Medical Sciences, The University of Sydney Sydney New South Wales Australia; ^12^ Department of Neurology Hanover Medical School Hanover Germany; ^13^ German Center for Neurodegenerative Diseases Munich Germany; ^14^ Edmond J. Safra Program in Parkinson's Disease University Health Network and the Division of Neurology, University of Toronto Toronto Canada; ^15^ Queen Square Brain Bank for Neurological Disorders, UCL Queen Square Institute of Neurology London United Kingdom; ^16^ Reta Lila Weston Institute of Neurological Studies UCL Queen Square Institute of Neurology London United Kingdom; ^17^ Department of Neurosciences Parkinson and Other Movement Disorders Center, University of California San Diego California USA; ^18^ Department of Neurology Mayo Clinic Rochester Minnesota USA; ^19^ Department of Neuropathology Institute of Brain Science, Hirosaki University Graduate School of Medicine Hirosaki Japan; ^20^ UCL Queen Square Institute of Neurology London United Kingdom; ^21^ Department of Uro‐Neurology The National Hospital for Neurology and Neurosurgery, Queen Square London United Kingdom; ^22^ Department of Medicine Surgery and Dentistry “Scuola Medica Salernitana”, Neuroscience Section, University of Salerno Salerno Italy; ^23^ Neurology, Internal Medicine Sakura Medical Center, Toho University Sakura Japan; ^24^ Parkinson's Disease and Movement Disorders Department HYGEIA Hospital, and Aiginiteion Hospital, University of Athens Athens Greece; ^25^ Philipps University Marburg, Germany and European University of Cyprus Nicosia Cyprus; ^26^ Centro de Investigación Biomédica en Red sobre Enfermedades Neurodegenerativas (CIBERNED) Hospital Clínic, IDIBAPS, Universitat de Barcelona Catalonia Spain; ^27^ Movement Disorders Unit, Neurology Service Hospital Clínic de Barcelona Catalonia Spain; ^28^ Department of Molecular Neurology The University of Tokyo, Graduate School of Medicine Tokyo Japan; ^29^ International University of Health and Welfare Chiba Japan

**Keywords:** multiple system atrophy, diagnostic criteria, diagnosis

## Abstract

**Background:**

The second consensus criteria for the diagnosis of multiple system atrophy (MSA) are widely recognized as the reference standard for clinical research, but lack sensitivity to diagnose the disease at early stages.

**Objective:**

To develop novel Movement Disorder Society (MDS) criteria for MSA diagnosis using an evidence‐based and consensus‐based methodology.

**Methods:**

We identified shortcomings of the second consensus criteria for MSA diagnosis and conducted a systematic literature review to answer predefined questions on clinical presentation and diagnostic tools relevant for MSA diagnosis. The criteria were developed and later optimized using two Delphi rounds within the MSA Criteria Revision Task Force, a survey for MDS membership, and a virtual Consensus Conference.

**Results:**

The criteria for neuropathologically established MSA remain unchanged. For a clinical MSA diagnosis a new category of clinically established MSA is introduced, aiming for maximum specificity with acceptable sensitivity. A category of clinically probable MSA is defined to enhance sensitivity while maintaining specificity. A research category of possible prodromal MSA is designed to capture patients in the earliest stages when symptoms and signs are present, but do not meet the threshold for clinically established or clinically probable MSA. Brain magnetic resonance imaging markers suggestive of MSA are required for the diagnosis of clinically established MSA. The number of research biomarkers that support all clinical diagnostic categories will likely grow.

**Conclusions:**

This set of MDS MSA diagnostic criteria aims at improving the diagnostic accuracy, particularly in early disease stages. It requires validation in a prospective clinical and a clinicopathological study. © 2022 The Authors. *Movement Disorders* published by Wiley Periodicals LLC on behalf of International Parkinson and Movement Disorder Society

## Graphical abstract

MDS Criteria for the Diagnosis of Multiple System Atrophy

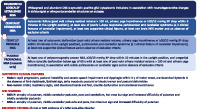

Multiple system atrophy (MSA) is a progressive neurodegenerative disease that clinically presents with autonomic failure, parkinsonism, and a cerebellar syndrome in various combinations and pathologically with glial cytoplasmic inclusions and neuronal loss predominantly in striatonigral and olivopontocerebellar systems.^1^ Three sets of diagnostic criteria for MSA have been proposed for clinical and research purposes in the past.^2^, ^3^, ^4^ The second consensus criteria for the diagnosis of MSA have been widely used as a diagnostic reference standard over the past 14 years.^3^ Data from two recent clinicopathological series, however, suggest that these have suboptimal accuracy (62%–79%) due to overlapping clinical manifestations with MSA look‐alike disorders.^5^, ^6^ Low sensitivity of MSA diagnosis at the first clinical visit (41% for possible and 18% for probable MSA) excludes many patients with early MSA from clinical trials of potential disease‐modifying drugs.^7^ Several issues associated with the suboptimal diagnostic performance of these criteria are outlined in a position statement by the International Parkinson and Movement Disorder Society (MDS) MSA Study Group.^8^ An early and reliable diagnosis of MSA remains an unmet need for patient care and counseling, for recruitment in clinical trials of disease‐modifying treatments, and for the development and validation of diagnostic tools.^8^ Thus, a MDS MSA Criteria Revision Task Force was convened to develop novel MSA diagnostic criteria with better accuracy, especially in early disease stages using evidence‐based and consensus‐based methodologies.

This review presents the new MDS diagnostic criteria for MSA (MDS MSA criteria) developed for clinical practice and research.

## Methodology of the Criteria Revision

The criteria were generated following the methodological requirements of transparency (provision of *a priori* methodological plan), reliability (use of reliable methodological tools), and multidisciplinarity (inclusion of all disciplines involved in the diagnostic process).

In 2018 the MSA Criteria Revision Task Force was appointed by MDS. The Oversight Committee and the Executive Team worked closely to define the scope and overall methodology of the project. Task Force experts were allocated according to their expertise into four working groups on clinical presentation (ataxia, autonomic failure non‐urogenital, parkinsonism, urogenital failure) and five working groups on diagnostic tests (autonomic function tests, imaging, other diagnostic tests, wet biomarkers, neuropathology). Working groups developed questions on clinical presentations and diagnostic tools relevant for the criteria revision. An extensive literature review was conducted to draft answers to the questions that served as an evidence base for the revision process. The Oversight Committee was in charge of drafting the criteria versions and proposing the revised draft version at each step. The Executive Team summarized the comments from the Delphi rounds, the Oversight Committee meetings, and the personal communication between the Task Force members. It also organized the virtual Consensus Conference, presented the results of the revision process during the Conference, and developed each draft criteria version together with the Oversight Committee.

A shared, transparent consensus‐building process leading to a final agreed version of the diagnostic criteria was ensured by the following three‐step approach.

First, the Task Force went through two Delphi rounds^9^ in February and July 2020. The first Delphi round (62 questions) aimed to analyze each element of the criteria in detail. It was framed according to the population–intervention–comparator–outcome (PICO) question model,^10^ and supplied with the quality of the evidence assessed using the validated tool.^11^ The second Delphi round (25 questions) was focused on the structure of the criteria and aimed to judge the elements that were new or that underwent major revision due to an unsatisfactory level of agreement during the first round. The agreement for deciding the solution or the reshaping of each element of the criteria was set to a level of ≥80% of the entire Task Force.

Second, all MDS members were asked to express their opinion on clarity and applicability of the criteria version developed after the second Delphi round, by means of an electronic survey (nine questions) in February 2021.^12^ The predefined level of acceptable agreement was set at ≥80% of responders. A total of 374 MDS members responded to at least one question and 242 (65%) completed the entire survey. Responders came from 78 different countries, and all continents were represented. Four topics of the criteria emerged with a suboptimal level of agreement (ranging from 65% to 77%): (1) terminology, aims and combination of features of the criteria levels; (2) the autonomic dysfunction core clinical features; (3) neuroimaging features; and (4) biomarkers.

Third, the final version of the criteria was established through a virtual Consensus Conference held over 2 days (April 30 and May 1, 2021) and achieved according to a simplified version of the standard procedure.^13^ On the first day, the four controversial issues were presented by the Executive Team and discussed by the whole Task Force. On the second day, in a closed session, the Consensus Development Panel established the final version of the criteria that were then announced in the concluding open session. After the virtual meeting, the Writing Committee drafted the document and circulated it to the Task Force members for final approval.

## MDS Diagnostic Criteria for MSA

The MDS criteria for the diagnosis of MSA define four levels of diagnostic certainty: neuropathologically established MSA, clinically established MSA, clinically probable MSA, and possible prodromal MSA. Neuropathologically established MSA replaces the category of definite MSA of the second consensus criteria, but the anchors remain unchanged. The diagnostic level of clinically established MSA is defined to respond to the need for diagnostic certainty at the clinical and patient level ensuring maximum specificity with acceptable sensitivity (Table 1). The category of clinically probable MSA is designed to balance sensitivity and specificity (Table 1). These categories are derived from the two clinical diagnostic levels of the second consensus criteria. A distinction between MSA‐parkinsonian type (MSA‐P) and MSA‐cerebellar type (MSA‐C) depending on the predominant motor phenotype is kept. The MDS MSA criteria introduce a new research category of possible prodromal MSA with very low specificity that is expected to continue to be refined with emerging data, particularly from prospective and biomarker studies (Table 2). All three clinical diagnostic categories need validation in future studies. Operationalized definitions of all features in the MDS MSA criteria are presented in the Lexicon (Table 3).

**TABLE 1:** Diagnostic criteria for clinically established and clinically probable multiple system atrophy

Division into clinically established MSA‐P or MSA‐C according to predominant motor syndrome			
**Essential features**	A sporadic, progressive adult (>30 years) onset disease		
	**Clinically established MSA**	**Clinically probable MSA**	
**Core clinical features**	Autonomic dysfunction defined as (at least one is required) Unexplained voiding difficulties with post‐void urinary residual volume ≥100 mL Unexplained urinary urge incontinence Neurogenic OH (≥20/10 mmHg blood pressure drop) within 3 minutes of standing or head‐up tilt test and at least one of Poorly L‐dopa‐responsive parkinsonism Cerebellar syndrome (at least two of gait ataxia, limb ataxia, cerebellar dysarthria, or oculomotor features)	At least two of: Autonomic dysfunction defined as (at least one is required): Unexplained voiding difficulties with post‐void urinary residual volume Unexplained urinary urge incontinence Neurogenic OH (≥20/10 mmHg blood pressure drop) within 10 minutes of standing or head‐up tilt test 2. Parkinsonism 3. Cerebellar syndrome (at least one of gait ataxia, limb ataxia, cerebellar dysarthria, or oculomotor features)	
**Supportive clinical (motor or non‐motor) features**	At least two	At least one^a^	
**MRI marker**	At least one	Not required	
**Exclusion criteria**	Absence	Absence	
**Supportive clinical features**			
**Supportive motor features**	Rapid progression within 3 years of motor onset	**Supportive non‐motor features**	Stridor
	Moderate to severe postural instability within 3 years of motor onset		Inspiratory sighs
	Craniocervical dystonia induced or exacerbated by L‐dopa in the absence of limb dyskinesia		Cold discolored hands and feet
	Severe speech impairment within 3 years of motor onset		Erectile dysfunction (below age of 60 years for clinically probable MSA)
	Severe dysphagia within 3 years of motor onset		Pathologic laughter or crying
	Unexplained Babinski sign		
	Jerky myoclonic postural or kinetic tremor		
	Postural deformities		
**MRI markers of clinically established MSA**			
Each affected brain region as evidenced by either atrophy or increased diffusivity counts as one MRI marker.			
For MSA‐P Atrophy of: Putamen (and signal decrease on iron‐sensitive sequences) Middle cerebellar peduncle pons Cerebellum “Hot cross bun” sign Increased diffusivity of: Putamen Middle cerebellar peduncle		For MSA‐C Atrophy of: Putamen (and signal decrease on iron‐sensitive sequences) Infratentorial structures (pons and middle cerebellar peduncle) “Hot cross bun" sign Increased diffusivity of: Putamen	
**Exclusion criteria**			
Substantial and persistent beneficial response to dopaminergic medications			
Unexplained anosmia on olfactory testing			
Fluctuating cognition with pronounced variation in attention and alertness and early decline in visuoperceptual abilities			
Recurrent visual hallucinations not induced by drugs within 3 years of disease onset			
Dementia according to DSM‐V within 3 years of disease onset			
Downgaze supranuclear palsy or slowing of vertical saccades			
Brain MRI findings suggestive of an alternative diagnosis (eg, PSP, multiple sclerosis, vascular parkinsonism, symptomatic cerebellar disease, etc.)			
Documentation of an alternative condition (MSA look‐alike, including genetic or symptomatic ataxia and parkinsonism) known to produce autonomic failure, ataxia, or parkinsonism and plausibly connected to the patient's symptoms			

^a^ Excluding erectile dysfunction as an isolated feature.

Abbreviations: MSA, multiple system atrophy; MSA‐P, MSA‐parkinsonian type; MSA‐C, MSA‐cerebellar type; OH, orthostatic hypotension; MRI, magnetic resonance imaging; DSM‐V, Diagnostic and Statistical Manual of Mental Disorders, Fifth Edition; PSP, progressive supranuclear palsy.

**TABLE 2:** Research criteria for possible prodromal multiple system atrophy

Essential features	A sporadic, progressive adult (>30 years) onset disease
**Clinical non‐motor features (entry criteria)**	At least one of the following: RBD (polysomnography proven) Neurogenic OH (≥20/10 mmHg blood pressure drop) within 10 minutes of standing or head‐up tilt Urogenital failure (erectile dysfunction in males below age of 60 years combined with at least one of unexplained voiding difficulties with post‐void urinary residual volume >100 mL and unexplained urinary urge incontinence)
**Clinical motor features**	At least one of the following: Subtle parkinsonian signs Subtle cerebellar signs
**Exclusion criteria**	Absence
**Exclusion criteria**	
At least one of unexplained anosmia on olfactory testing or abnormal cardiac sympathetic imaging (^123^I‐MIBG‐scintigraphy)	
Fluctuating cognition with pronounced variation in attention and alertness and early decline in visuoperceptual abilities	
Recurrent visual hallucinations not induced by drugs within 3 years of disease onset	
Dementia according to DSM‐V within 3 years of disease onset	
Downgaze supranuclear gaze palsy or slowing of vertical saccades	
Brain MRI findings suggestive of an alternative diagnosis (eg, PSP, multiple sclerosis, vascular parkinsonism, symptomatic cerebellar disease, etc.)	
Documentation of an alternative condition (MSA look‐alike, including genetic or symptomatic ataxia and parkinsonism) known to produce autonomic failure, ataxia, or parkinsonism and plausibly connected to the patient's symptoms	

Abbreviations: MSA, multiple system atrophy; RBD, rapid eye movement  sleep behavior disorder; OH, orthostatic hypotension; DSM‐V, Diagnostic and Statistical Manual of Mental Disorders, Fifth Edition; MRI, magnetic resonance imaging; PSP, progressive supranuclear palsy.

**TABLE 3:** Lexicon with operationalized definitions of features of the Movement Disorders Society criteria for the diagnosis of multiple system atrophy

Feature	Operationalized definition
Disease onset	First subjective complaint of symptoms related to MSA
**Clinically established and clinically probable MSA**	
**Core autonomic features**	
Unexplained voiding difficulties with post‐void urinary residual volume >100 mL (for clinically established MSA) or post‐void urinary residual of any volume (for clinically probable MSA)	Voiding difficulties with >100 mL of urine (for clinically established MSA) or any volume of urine (for clinically probable MSA) retained in the bladder after a voluntary void measured by bladder ultrasound, urodynamics, or in‐out catheterisation. Secondary causes such as bladder outflow obstruction due to prostate enlargement should be excluded.
Unexplained urinary urge incontinence	Complaint of involuntary urine leakage associated with urgency in the absence of urinary tract infections. Non‐neurogenic causes such as previous pelvic surgery or pelvic floor prolapse should be excluded.
Neurogenic OH	≥20 mmHg systolic BP drop usually accompanied by a diastolic BP drop of ≥10 mmHg and ΔHR/ΔSBP ratio < 0.5 bpm/mmHg within 3 minutes (for clinically established MSA) or within 10 minutes (for clinically probable MSA) of standing or head up tilt using oscillometric measurements. Secondary causes such as diabetic autonomic neuropathy should be excluded. Medications that impair the HR response to orthostasis (such as beta blockers) should be excluded.
**Core parkinsonian features**	
Parkinsonism	Presence of bradykinesia plus rigidity or tremor (excluding intentional tremor in a patient with cerebellar syndrome) judged by a movement disorder specialist after examination carried out as described in the MDS‐UPDRS III; bradykinesia = slowness of movement and decrement in amplitude or speed (or progressive hesitations or halts) as movements are continued; rigidity = velocity‐independent resistance to passive movement not solely reflecting failure to relax that may be accompanied by cogwheel phenomenon; tremor = rhythmic or arrhythmic involuntary movement in arms or legs.
Poor L‐dopa responsiveness (for clinically established MSA)	Defined by history or as <30% improvement on the MDS‐UPDRS III on up to 1000 mg L‐dopa as needed or tolerated for at least a month as judged by a movement disorder specialist.
**Core cerebellar features**	
Cerebellar syndrome	At least two (for clinically established) or at least one (for clinically probable MSA) of gait ataxia, limb ataxia, cerebellar dysarthria, or oculomotor dysfunction; oculomotor features = sustained nystagmus (gaze‐evoked horizontal or downbeat) or saccadic hypermetria.
**Supportive motor features**	
Rapid progression within 3 years of motor onset	Needs help with some chores or greater disability within 3 years of motor onset assessed by history. Rate of progression is rapid in comparison to what a movement disorder specialist would anticipate for Parkinson's disease.
Moderate to severe postural instability within 3 years of motor onset	Deficient postural response defined as at least three steps backwards or tendency to fall if not caught by examiner upon pull test within 3 years of motor onset.
Craniocervical dystonia induced or exacerbated by L‐dopa in the absence of limb dyskinesia	Involuntary dystonic movements of the face induced or exacerbated by L‐dopa in the absence or presence of very mild limb dyskinesia.
Severe speech impairment within 3 years of motor onset	Slow, slurred, or dysphonic speech severe enough to require occasional repetition of statements during interview within 3 years of motor onset.
Severe dysphagia within 3 years of motor onset	Unexplained difficulty while drinking or eating severe enough to request dietary adaptations within 3 years of motor onset.
Unexplained Babinski sign	Other causes such as mass lesions, vascular, demyelinating, metabolic diseases, cervical myelopathy, and infections should be excluded.
Jerky myoclonic postural or kinetic tremor	Irregular small‐amplitude postural or kinetic tremor of the hands or fingers with stimulus‐sensitive myoclonus.
Postural deformities	At least one of disproportionate anterocollis or laterocollis, camptocormia, Pisa syndrome, or contractures of hands or feet (excluding Dupuytren's or contracture due to other known cause including corticobasal syndrome); disproportionate anterocollis or laterocollis = marked neck anteroflexion or lateroflexion, may be partially overcome by voluntary or passive movement, camptocormia = severe anterior flexion of the spine, Pisa syndrome = severe lateral flexion of the spine.
**Supportive non‐motor features**	
Stridor	High‐pitched inspiratory breathing sound emitted during sleep or while awake. Laryngoscopy could be considered to exclude mechanical lesions or functional vocal cord abnormalities related to other neurological disorders.
Inspiratory sighs	Involuntary deep inspiratory sighs or gasps.
Cold discolored hands and feet	Newly developed coldness and colour change (purple or blue) with blanching on pressure and poor circulatory return.
Erectile dysfunction (below age of 60 years for clinically probable MSA)	Persistent inability to achieve or maintain an erection sufficient to engage in sexual activity (below age of 60 years for clinically probable MSA).
Pathologic laughter or crying	Emotional incontinence not necessary to be witnessed by clinician.
**MRI markers**	
MRI markers (for clinically established MSA)	Structural brain MRI (1.5 or 3.0 T) analysis is based on visual inspection by a neuroradiologist who has explicitly to be advised by a movement disorder specialist to evaluate these features. Diffusion brain MRI analysis is based on quantitative assessments by a neuroradiologist who has explicitly to be advised by a movement disorder specialist to evaluate these features.
**Possible prodromal MSA**	
**Clinical non‐motor features (entry criteria)**	
PSG‐proven REM sleep behavior disorder	According to American Academy of Sleep Medicine's *International Classification of Sleep Disorders*, *Third Edition* (ICSD‐3).
Neurogenic OH	≥20 mmHg systolic BP drop usually accompanied by a diastolic BP drop of ≥10 mmHg and ΔHR/ΔSBP ratio < 0.5 bpm/mmHg within 10 minutes of standing or head up tilt using oscillometric measurements. Secondary causes such as diabetic autonomic neuropathy should be excluded. Medications that impair the HR response to orthostasis (such as beta blockers) should be excluded.
Erectile dysfunction in males below age of 60 years combined with at least one of unexplained voiding difficulties with post‐void urinary residual volume >100 mL and unexplained urinary urge incontinence	Persistent inability to achieve or maintain an erection sufficient to engage in sexual activity in males below age of 60 years combined with at least one of unexplained voiding difficulties with >100 mL of urine retained in the bladder after a voluntary void measured by bladder ultrasound, urodynamics, or in‐out catheterization and complaint of involuntary urine leakage associated with urgency in the absence of urinary tract infections. Secondary causes of post‐void urinary residual such as bladder outflow obstruction due to prostate enlargement and non‐neurogenic causes of urinary urge incontinence such as previous pelvic surgery or pelvic floor prolapse should be excluded.
**Clinical motor features**	
Subtle parkinsonian signs	Presence of parkinsonian motor signs not satisfying MDS Parkinson's disease diagnostic criteria^33^ for parkinsonism, judged as subtle by a movement disorder specialist, and not requiring dopaminergic medications.
Subtle cerebellar signs	At least one of impaired tandem gait or gait ataxia, limb ataxia, cerebellar dysarthria, or oculomotor features, judged as subtle by a movement disorder specialist.
**Exclusion criteria for all categories (if not indicated differently)**	
Substantial and persistent beneficial response to dopaminergic medications (applicable for clinically established and clinically probable MSA)	As judged by a movement disorder specialist.
Unexplained anosmia on olfactory testing	Not explained by other common causes such as allergic rhinitis or smoking, nasal structural lesions, or nasal surgery.
Abnormal cardiac sympathetic imaging (^123^I‐MIBG‐scintigraphy) (applicable for possible prodromal MSA category)	Abnormal heart/mediastinum ratio 4 hours after intravenous injection of ^123^I‐MIBG, as assessed by a nuclear medicine specialist. Medications affecting noradrenaline transporter and vesicular storage, structural heart disease, and common causes of small fiber neuropathies, such as diabetes mellitus that may affect the findings must be excluded.
Fluctuating cognition with early decline in visuoperceptual abilities	Fluctuating cognition with pronounced variation in attention and alertness and early decline in visuoperceptual abilities.
Recurrent visual hallucinations	Not induced by drugs within 3 years of disease onset.
Dementia	According to DSM‐V within 3 years of disease onset.
Downgaze supranuclear palsy	Downgaze supranuclear palsy or slowing of vertical saccades.
Brain MRI findings suggestive of an alternative diagnosis	For example, PSP, multiple sclerosis, vascular parkinsonism, symptomatic cerebellar disease.
Documentation of an alternative condition known to produce autonomic failure, ataxia, or parkinsonism and plausibly connected to the patients' symptoms	MSA look‐alike, including genetic or symptomatic ataxia and parkinsonism.

Abbreviations: MSA, multiple system atrophy; OH, orthostatic hypotension; BP, blood pressure; HR, heart rate; SBP, systolic blood pressure; bpm, beats per minute; MDS‐UPDRS III, Movement Disorder Society‐Sponsored Revision of the Unified Parkinson's Disease Rating Scale Part III; MRI, magnetic resonance imaging; PSG, polysomnography; PSP, progressive supranuclear palsy.

## Neuropathologically Established MSA

Neuropathologically established MSA corresponds to the definite MSA category of the second consensus criteria. Neuropathologic findings of widespread and abundant central nervous system (CNS) α‐synuclein‐positive glial cytoplasmic inclusions in association with neurodegenerative changes in striatonigral or olivopontocerebellar structures characterize MSA on autopsy.^14^

## Essential Features of Clinical MSA Diagnosis

As in the second consensus criteria, prerequisites for a clinical diagnosis of MSA for all levels of certainty include symptom onset after 30 years of age (since there are no postmortem‐proven MSA cases with onset in the third decade or earlier), a negative family history, and a progressive disease course.

## Clinically Established MSA

**FIG. 1:**
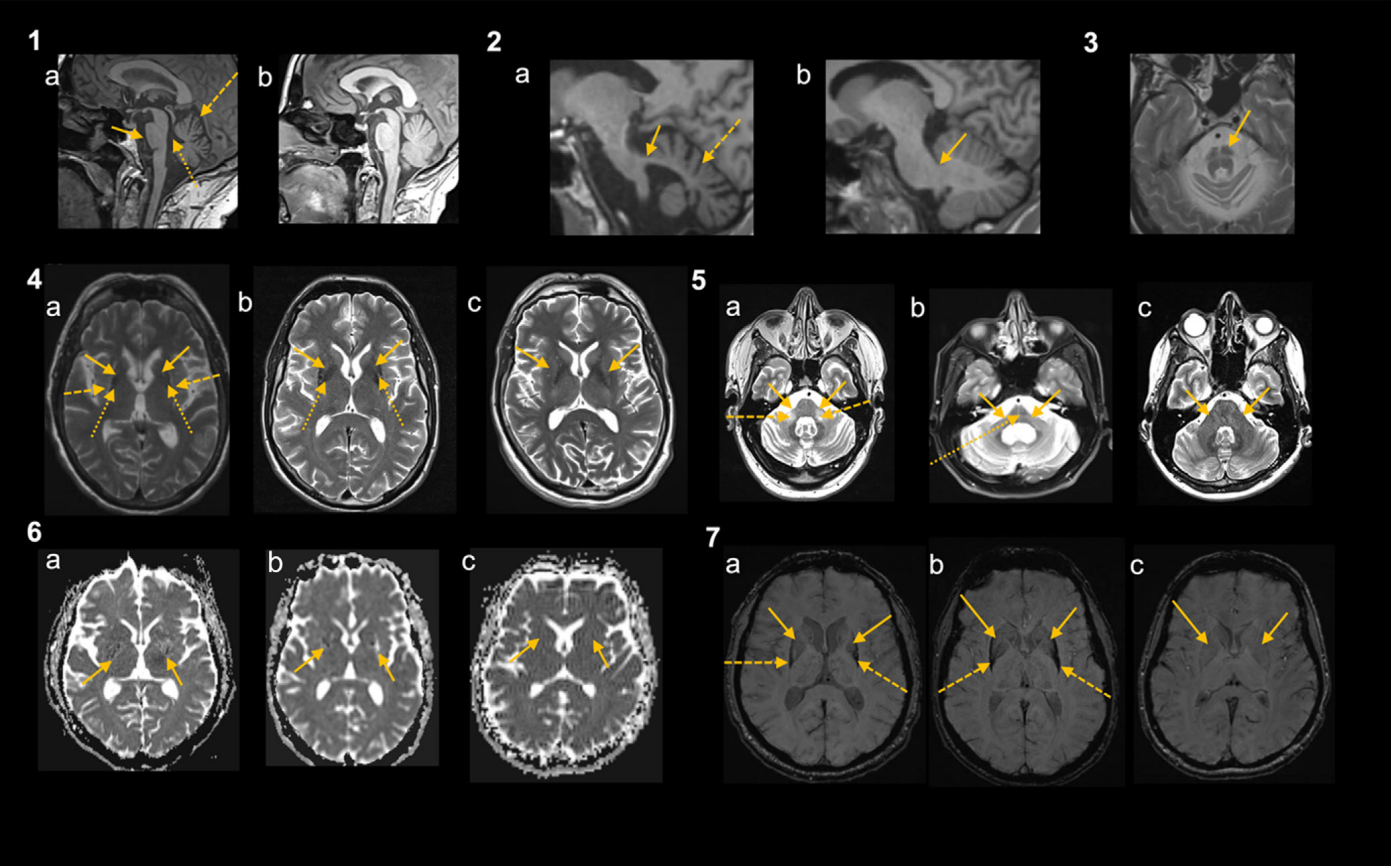
Brain magnetic resonance imaging (MRI) markers of clinically established multiple system atrophy (MSA) (reprinted from Fancuilli et al^81^ © 2019 Elsevier Inc.) Panel 1: midsagittal T1‐weighted images showing infratentorial atrophy including pontine atrophy (solid arrow) and cerebellar atrophy with enlarged fissures and interfolial spaces of the cerebellum (dashed arrow) and consecutive dilated forth ventricle (dotted arrow) in a patient with MSA (a), while there is no relevant infratentorial atrophy in a patient with Parkinson's disease (PD) (c). Panel 2: parasagittal T1‐weighted images showing middle cerebellar peduncles (MCP) atrophy between the peripeduncular cerebrospinal fluid spaces of pontocerebellar cisterns (solid arrow) in a patient with MSA (a), while there is no MCP atrophy (solid arrow) in a patient with PD (b). Moreover, there is cerebellar atrophy with enlarged fissures and interfolial spaces of the cerebellum (dashed arrow) in the patient with MSA (a) compared to the patient with PD (b). Panel 3: “hot cross bun” sign (arrow) in a patient with MSA on T2‐weighted images. Panel 4: putaminal atrophy (solid arrows) (a, b) and signal changes including hyperintense rim (dashed arrows) (a) and putaminal hypointensity in comparison with the globus pallidus (dotted lines) (a, b) at both sides in patients with MSA (a, b) on T2‐weighted images compared to a patient with PD (c) having no putaminal atrophy (arrows). Panel 5: atrophy of MCP (solid arrows) on T2‐weighted images (a, b) with MCP‐sign (hyperintensity in the MCP) (dashed arrows) (a) and “hot cross bun” sign (dotted arrow) (b) in patients with MSA (a, b) compared to a PD patient with normal MCP (solid arrows) (c). Panel 6: note the diffuse hyperintensity (corresponding to increased diffusivity values) in the posterior part of both putamina (solid arrows) in patients with MSA (a, b) compared to a PD patient with no diffusivity changes in the putamen (solid arrows) (c) on diffusion imaging. The changes in the MSA patient (a) were observed only 6 months after onset of levodopa‐responsive parkinsonism with an anticipation of 18 months in relation to the clinical diagnosis of possible MSA and of 24 months for the diagnosis of probable MSA. Panel 7: putaminal atrophy can also be determined with iron‐sensitive sequences as demonstrated in these images. Putaminal atrophy (solid arrows) and putaminal hypointensity (ie, signal decrease) (dashed arrows) on susceptibility weighted imaging in a patient with MSA (a, b) compared to a PD patient with no putaminal atrophy (solid lines) (c). As in these MSA patients, putaminal hypointensity starts typically in the dorsolateral part of the putamen. [Color figure can be viewed at wileyonlinelibrary.com]

Clinically established MSA is defined as a combination of core clinical features, at least two supportive motor or non‐motor features, at least one brain magnetic resonance imaging (MRI) marker suggestive of MSA (Fig. 1), and a lack of exclusion criteria (Table 1, explained in Table 3). The presence of at least one core feature of voiding difficulties with post‐void residual volume (PVR) >100 mL, urinary urge incontinence or neurogenic orthostatic hypotension (nOH) within 3 minutes of standing, or head up tilt (HUT) associated with either levodopa‐unresponsive parkinsonism or cerebellar syndrome, or both, secures high diagnostic specificity of this category. The diagnosis of clinically established MSA requires brain MRI markers that are specific, but, similarly to the core clinical features, often manifest later in the disease course. If clinical criteria for clinically established MSA are fulfilled but MRI marker is lacking, the patient should be diagnosed with clinically probable MSA. Biomarkers should not be used to support the diagnosis of clinically established MSA in brain MRI‐negative cases; instead, their role remains subject to future research (see later).

## Clinically Probable MSA

A diagnosis of clinically probable MSA requires at least two core features of autonomic failure (voiding difficulties with PVR of any volume, urinary urge incontinence and delayed nOH within 10 minutes of standing, or HUT), parkinsonism (irrespective of response to levodopa), and cerebellar impairment, in any combination, including parkinsonism combined with cerebellar signs without autonomic failure. Core clinical features of clinically probable MSA are more sensitive and usually manifest earlier compared to the core features of clinically established MSA. When associated with at least one supportive motor or non‐motor clinical feature, excluding erectile dysfunction (ED) due to its low specificity, the balanced sensitivity and specificity of this category is secured. Brain MRI markers are not required for the diagnosis of clinically probable MSA. In brain MRI‐positive MSA patients that manifest with clinical features of clinically probable MSA, the diagnosis of clinically probable MSA remains.

## Core Clinical Features of Clinically Established and Clinically Probable MSA

### Urogenital Failure

Lower urinary tract (LUT) symptoms suggestive of urogenital failure are the sole initial manifestation of MSA in 18% of patients, with a mean onset of 2.8 years prior to the onset of motor symptoms.^15^ In contrast, LUT dysfunction is highly uncommon in early stages of Parkinson's disease (PD) and sporadic adult onset ataxia (SAOA). The following assessments are recommended when screening for LUT dysfunction in patients with suspected MSA at the initial assessment: (1) history taking for the evaluation of urinary storage symptoms (urinary urgency, daytime frequency, nocturia, and urge incontinence; collectively called “overactive bladder symptoms“) and voiding symptoms (hesitancy, intermittent urinary stream or poor flow, sensation of incomplete bladder emptying, double voiding); (2) completing a 3‐day bladder diary that provides assessment of LUT symptoms, and is the only assessment for nocturnal polyuria, which may occur in MSA;^16^ (3) digital rectal examination and ultrasonography to evaluate possible enlargement of the prostate gland as a contributor to LUT symptoms in the male patient with suspected MSA; and (4) measurement of the PVR, preferably sonographically, alternatively by urodynamics or in‐out catheterization.

As disease advances, patients with MSA demonstrate progressive increase in the PVR, whereas patients with PD and SAOA usually develop overactive bladder symptoms.^17^ In one large study, PVR in patients with MSA increased from 71 mL in the first year to 129 mL in the second year from disease onset.^17^ Significantly elevated PVR is the most specific sign of bladder dysfunction in MSA versus PD (sensitivity: 34%, specificity: 95%), but is not useful to distinguish MSA from progressive supranuclear palsy (PSP).^17^, ^18^, ^19^, ^20^ An arbitrary cutoff at >100 mL PVR volume is set to secure high specificity of clinically established diagnosis. A lower PVR is allowed for clinically probable diagnosis, as this finding is still distinctive of MSA in the earliest disease stages. Compared to the PVR, urinary urge incontinence is more sensitive but less specific for the diagnosis of MSA (sensitivity: 48%, specificity: 34%).^20^ Thus, urinary urge incontinence features in the diagnosis of both clinically established and clinically probable MSA.

The onset of ED preceded the onset of bladder symptoms in 58% of men with probable MSA diagnosed according to the second consensus criteria.^21^ Since ED is common in the general population and elderly patients with MSA look‐alike disorders, it needs to be associated with at least one more supportive clinical feature for a diagnosis of clinically established MSA. Diagnosis of clinically probable MSA may be supported only if ED manifests before the age of 60 years and is associated with another supportive clinical feature of MSA.

### Cardiovascular Autonomic Failure

Neurogenic orthostatic hypotension is defined as a ≥20 mmHg systolic blood pressure (SBP) drop usually accompanied by a diastolic BP (DBP) drop of ≥10 mmHg and a Δheart rate (HR)/ΔSBP ratio < 0.5 beats per minute (bpm)/mmHg within 3 minutes of standing or HUT using oscillometric measurements.^22^, ^23^ Medications that impair the HR response to orthostasis (such as beta blockers) should be excluded. To determine a neurogenic cause of OH, it is essential to identify the blunted HR increase during hypotension and exclude common secondary causes.^22^ The classical ≥20/10 mmHg BP drop within 3 minutes in the upright position criterion for nOH has better sensitivity for the diagnosis of MSA compared to the ≥30/15 mmHg BP drop criterion (the one used in the second consensus criteria) with similar specificity (≥20/10 mmHg, sensitivity: 46% and specificity: 76%; ≥30/15 mmHg sensitivity: 28% and specificity: 80%).^20^ Both the SBP drop of at least 20 mmHg and the DBP drop of at least 10 mmHg were fulfilled by only 49.5% of patients with nOH in one study.^24^ Since 95% of patients with nOH can be identified by a SBP criterion alone (an isolated fall in DBP is rare in these patients),^24^ we allow a diagnosis of nOH if only the systolic criterion is fulfilled. The DBP drop criterion is non‐specific and should not be used in isolation. Sensitivity of nOH within 3 minutes of standing or HUT is modest to moderate at early stages of MSA and increases with disease duration (for MSA‐P vs. PD sensitivity: 20%–61%, specificity: 69%–100%, for MSA‐C vs. SAOA sensitivity: 32%–56%, specificity: 94%–100%).^25^, ^26^, ^27^, ^28^, ^29^ Therefore, to increase diagnostic sensitivity, delayed nOH occurring after 3 minutes but within 10 minutes in the upright position is included as a feature of clinically probable MSA.^30^

Continuous non‐invasive HR and BP monitoring may be used for the diagnosis of nOH in the centers where these facilities are available.^31^ The reference standard to ascertain the neurogenic nature of OH is evaluating the BP recovery time with the Valsalva maneuver. A prolonged BP recovery time indicates peripheral sympathetic adrenergic failure.^32^

### Parkinsonism

Bradykinesia associated with rigidity or tremor (excluding intentional tremor in a patient with cerebellar syndrome), ascertained by a movement disorder specialist, defines parkinsonism in MSA.^33^ A beneficial response to levodopa in 42.5%–56.7% of MSA‐P patients and 12.9%–25% of MSA‐C patients associated with motor fluctuations and dyskinesia that lasted for a mean of 3.5 ± 2.7 years and 3.2 ± 2.3 years was reported in the prospective European and the US natural history studies, respectively.^34^, ^35^ Poor levodopa responsiveness, defined by history or as <30% improvement on the MDS‐Sponsored Revision of the Unified Parkinson's Disease Rating Scale Part III (MDS‐UPDRS III) on 1000 mg L‐dopa if tolerated or required (Table 3), is needed for a clinically established diagnosis, while moderate, good, or excellent response qualifies the patient as clinically probable MSA. Acute levodopa challenge test cannot reliably assist in the early diagnosis of MSA, and a negative test result should not discourage clinicians from an initiation of chronic levodopa treatment.^36^

### Cerebellar Syndrome

Cerebellar syndrome is defined as at least two of gait ataxia, limb ataxia, cerebellar dysarthria, or cerebellar oculomotor dysfunction (sustained gaze‐evoked horizontal or positional downbeat nystagmus and saccadic hypermetria) for the diagnosis of clinically established MSA, whereas at least one of these features is sufficient for the diagnosis of clinically probable MSA. In order to raise sensitivity, impaired tandem gait is included as a clinical feature of possible prodromal MSA, in addition to other cerebellar signs, ascertained as subtle by a movement disorder specialist (Table 3).

## Supportive Motor and Non‐Motor Features of Clinically Established and Clinically Probable MSA

Supportive clinical features for MSA diagnosis (Table 1, explained in Table 3) and MSA red flags are synonymous. An accumulation of supportive clinical features over a lifetime increases the accuracy of a postmortem‐confirmed MSA diagnosis.^5^ The European MSA Study Group reported that the presence of multiple supportive clinical features may increase sensitivity of MSA diagnosis.^37^ However, there is no difference in the frequency of supportive clinical features for MSA within 3 years from disease onset between postmortem‐confirmed cases with MSA and MSA look‐alike disorders, including PD, dementia with Lewy bodies, and PSP.^5^

Compared to PD, postural instability within the first 3 years of disease is suggestive of MSA (sensitivity: 27%–45%, specificity: 88.7%), but is even more specific if occurs in the first year from motor onset (sensitivity: 23.5%, specificity: 94%).^7^, ^38^ Median latency to falls was shorter in MSA (24 months) than in PD (118 months).^39^ A high likelihood of postmortem MSA diagnosis (96.2% for MSA‐P and 86% for MSA‐C) as opposed to Lewy body disorder (LBD) diagnosis was reported if at least one of orofacial dystonia, inspiratory sighs, contractures of hands or feet, polyminimyoclonus, severe dysarthria, pathologic laughter or crying, and cold hands and feet (for each supportive clinical feature specificity >90%) manifested during a lifetime.^5^ A classic pill‐rolling tremor is infrequent in MSA.^40^

The MSA supportive clinical features were originally described to distinguish MSA from PD, but these features (orofacial dystonia, inspiratory sighs, contractures of hands or feet, jerky myoclonic postural or action tremor, polyminimyoclonus, severe dysphonia, snoring) may also be useful for differentiation from PSP.^5^ In addition, if early and severe autonomic failure occurs within 3 years from symptom onset the patient is 3.4 times more likely to be diagnosed with MSA instead of PSP.^5^ Thus, in a patient with early axial motor signs, severe autonomic failure is helpful to guide a diagnosis towards MSA.

Non‐motor features including urinary urgency and incontinence (sensitivity: 58%, specificity: 100% at initial assessment), ED (sensitivity: 81%, specificity: 100% at initial assessment), nOH (≥20/10 mmHg BP drop) (sensitivity: 56%, specificity: 100% at initial assessment), rapid eye movement sleep behavior disorder (RBD) (sensitivity: 43%, specificity: 100% for clinically ascertained RBD within 4 years of disease onset), and stridor in a patient with ataxia are suggestive of MSA‐C after exclusion of the most common genetic mimics.^28^, ^41^, ^42^, ^43^ However, RBD is considered as a research biomarker for MSA diagnosis due to its different weighing for the diagnosis of MSA‐P (ie, high prevalence in other synucleinopathies). Stridor is distinctive of both motor subtypes of MSA compared to their mimics and, as such, is considered a supportive non‐motor feature.^44^

## Brain MRI Markers

At least one structural brain 1.5 T or 3.0 T MRI marker of atrophy or diffusivity changes in the putamen or infratentorial structures suggestive of MSA is necessary for the diagnosis of clinically established MSA (Table 1, Fig. 1). MRI analysis, including diffusion images, should be based on visual inspection by a neuroradiologist who has explicitly been advised by a movement disorder specialist to evaluate these features. Atrophy of the putamen (and signal decrease on iron‐sensitive sequences corresponding to increased iron content), pons, middle cerebellar peduncles (MCP) and cerebellum, the “hot‐cross bun” sign (cruciform hyperintensity in the pons on T2 images), and increased diffusivity in the putamen and MCP are radiological hallmarks of clinically established MSA‐P (Fig. 1). For the diagnosis of clinically established MSA‐C, at least one of atrophy or increased diffusivity of putamen, atrophy of the pons or MCP, or the “hot‐cross bun” sign are required. An isolated atrophy of the cerebellum or increased diffusivity in the MCP do not support the diagnosis of clinically established MSA‐C due to overlaps with sporadic and genetic disorders presenting with cerebellar atrophy (eg, paraneoplastic or genetic ataxia) (Table 1).

In postmortem‐confirmed MSA cases the overall accuracy of radiological MSA diagnosis based on conventional MRI was 76.9%.^45^ Structural brain abnormalities have excellent specificity for distinguishing MSA from PD, but unsatisfactory sensitivity, particularly in early stages (for accuracy values of each affected brain region see Pellecchia et al^46^). Signal abnormalities are influenced by the applied magnetic field strength. The putaminal rim sign (hyperintense signal in the dorsolateral margin of the putamen on T2 images) is omitted from the current criteria proposal because it is a common finding in healthy subjects when using a 3.0 T MRI and has limited differential diagnostic potential in separating MSA from PSP.^47^ The “hot cross bun” sign has been described in non‐degenerative parkinsonism and spinocerebellar ataxia type 2 and 3.^48^, ^49^

Differences in putaminal diffusivity show excellent accuracy for distinguishing MSA from PD (sensitivity: 90%, specificity: 93%), while increased diffusivity of the MCP combined with normal diffusivity of the superior cerebellar peduncles is useful for separating MSA from PSP (sensitivity: 91%, specificity: 84%).^46^, ^50^

Multimodal MRI approaches that combine volumetric measurements, diffusion‐weighted imaging, and iron‐sensitive sequences for the development of diagnostic algorithms as well as novel post‐processing methods of volumetric images resulting in automated volume segmentation on the single‐patient level, have reported good to excellent accuracy for the diagnosis of MSA, but are currently restricted to specialized research centers.^51^, ^52^, ^53^ Harmonization of MRI protocols and analysis platforms and their validation in prospective cohorts will be required to enable the widespread use of advanced MRI methods.

## Exclusion Features for Clinically Established and Clinically Probable MSA Diagnosis

Some of the exclusion features (Table 1, explained in Table 3) such as persistent levodopa responsiveness,^34^, ^35^ hyposmia,^54^ cognitive impairment, and hallucinations^55^ may occasionally manifest in MSA, but are outlined as features excluding MSA diagnosis to secure the diagnostic specificity. Structural brain MRI should be performed in all patients to exclude findings suggestive of an alternative diagnosis. Alternative conditions known to produce autonomic failure, ataxia, or parkinsonism (including genetic^56^ and symptomatic parkinsonism and ataxia) and plausibly connected to the patient symptoms need to be excluded for all MSA diagnostic categories. Genetic screening for the common spinocerebellar ataxia (SCA1, SCA2, SCA3, SCA6, SCA7, SCA12, and SCA17), FRDA, FXTAS, and CANVAS should be considered especially in patients with cerebellar features.^57^

## Possible Prodromal MSA

The possible prodromal MSA category has been devised to capture MSA patients at the earliest prodromal stage when symptoms and signs are present, but yet are insufficient for the diagnosis of clinically established and clinically probable MSA. This is a research category with limited specificity, which needs validation in future prospective studies. It is composed of essential features, clinical non‐motor and motor features, and a lack of exclusion criteria (Table 2, explained in Table 3). Either polysomnography (PSG)‐proven RBD or isolated autonomic failure, defined as at least one of urogenital failure with PVR >100 mL or urinary urge incontinence, or nOH within 10 minutes of standing or HUT, are the current entry criteria (clinical non‐motor features) for a diagnosis of possible prodromal MSA. Among patients with idiopathic RBD who develop neurodegenerative disease after 4–5 years, 8% were eventually diagnosed as probable MSA according to the second consensus criteria.^58^ Conversion rate to MSA ranged between 8% and 28% and occurred after 2 to over 10 years of isolated autonomic failure. Predictors of the phenoconversion were supine norepinephrine >100 pg/mL, preserved olfaction, supine HR >70 bpm, age of onset in the early 50s, orthostatic HR increase >10 bpm within 3 minutes, Composite Autonomic Severity vagal score <2, preganglionic sweat loss pattern, and subtle motor signs not qualifying for parkinsonism or ataxia at initial assessment.^59^, ^60^, ^61^

Presence of subtle parkinsonian motor signs (insufficient to satisfy MDS diagnostic criteria for PD^33^) or cerebellar signs (clinical motor features) is necessary for the diagnosis of possible prodromal MSA. Subtle motor signs are useful for distinguishing patients with possible prodromal MSA from patients with pure autonomic failure. There is a lack of exclusion criterion on levodopa responsiveness, because these patients have mild parkinsonian signs that do not require dopaminergic treatment. Since presence of mild parkinsonian signs in a patient with PSG‐proven RBD may point towards the diagnosis of LBD, it is mandatory to exclude at least one of unexplained anosmia on olfactory testing or abnormal cardiac sympathetic imaging. Supportive research biomarkers for possible prodromal MSA are similar to those for clinically established and clinically probable MSA categories, with an exception of PSG‐proven RBD that is an essential feature of this category.

It is acknowledged that these new criteria for possible prodromal MSA are limited to patients who develop early autonomic dysfunction or RBD and exclude those who present with early motor or other non‐motor features in the absence of these. Future research involving diagnostic biomarkers (see later) will certainly expand this category to include patients with a broader range of presenting features.

## Supportive Biomarkers for MSA Diagnosis

A list of research biomarkers for MSA diagnosis consists of supportive investigational tools that provide laboratory findings suggestive of MSA, but are not required for the MSA diagnosis (Table 4). They are not formally included in the MDS MSA criteria due to their limited availability (according to the feedback from the MDS membership via survey), suboptimal diagnostic accuracy, or lack of diagnostic validation (particularly for new developments such as CSF α‐synuclein oligomers). Evidence proving the value of supportive biomarkers in possible prodromal MSA is limited. However, if available, to provide more evidence for future studies, research biomarkers should be applied to all MSA diagnostic categories. The Task Force recognizes that the list of supportive biomarkers will likely grow when more data become available and that criteria refinement will be needed. Task Force will plan the systematic identification of emerging or consolidating diagnostic biomarkers to promptly advise the update of the MSA criteria. For example, laryngeal motion abnormalities were detected in 93% of patients with MSA compared to 1.8% of patients with PD using flexible fiberoptic video rhinolaryngoscopy evaluation of a swallowing task.^62^ The so‐called horizon scanning methodology,^63^ a tool adopted globally to identify, assess, and prioritize innovations at an early stage of their development, is proposed to achieve this goal.

**TABLE 4:** Supportive biomarkers suggestive of multiple system atrophy (MSA) but not required for the MSA diagnosis with operationalized definitions (applicable for all clinical MSA diagnostic categories if not indicated otherwise)

Supportive biomarker		Operationalized definition
MRI markers^a^		
Atrophy of: Putamen (and signal decrease on iron‐sensitive sequences) Middle cerebellar peduncle Pons Cerebellum “Hot cross bun” sign Increased diffusivity of: Putamen Middle cerebellar peduncle		Structural brain MRI (1.5 or 3.0 T) analysis is based on visual inspection by a neuroradiologist who has explicitly to be advised by a movement disorder specialist to evaluate these features. Diffusion brain MRI analysis is based on quantitative assessments by a neuroradiologist who has explicitly to be advised by a movement disorder specialist to evaluate these features.
FDG‐PET markers^b^		
For MSA‐P hypometabolism of: Putamen Brainstem Cerebellum	For MSA‐C hypometabolism of: Putamen	Based on visual inspection by a nuclear medicine specialist.
Normal cardiac sympathetic imaging (^123^I‐MIBG‐scintigraphy)^c^		Normal heart/mediastinum ratio 4 hours after intravenous injection of ^123^I‐MIBG, as assessed by a nuclear medicine specialist. Medications affecting noradrenaline transporter and vesicular storage, structural heart disease, and common causes of small fiber neuropathies, such as diabetes mellitus that may affect the findings must be excluded.
PSG‐proven REM sleep behavior disorder^c^		According to American Academy of Sleep Medicine's *International Classification of Sleep Disorders*, *Third Edition* (ICSD‐3).
Supine plasma norepinephrine level >100 pg/mL associated with neurogenic OH		Using high‐performance liquid chromatography with electrochemical detection after 10 minutes lying supine associated with neurogenic OH.
Detrusor hyperactivity with impaired contraction or detrusor sphincter dyssynergia on urodynamic testing		Synchronous contraction of detrusor and urethral sphincter during voiding on urodynamic study.
Unexplained abnormal sphincter EMG		Concentric needle EMG of external anal sphincter demonstrating more than 20% of MUPs having a duration >10 ms, or the average duration of MUPs >10 ms. Recorded MUPs should be analyzed manually to include late components. Similar changes of chronic reinnervation may be seen with cauda equina injury, following pelvic surgery and obstetric pelvic floor tears and other neurodegenerative disorders (such as PSP and long standing PD).
CSF α‐synuclein oligomers detected by PMCA or RT‐QuIC		Detected by PMCA or RT‐QuIC.
Increased plasma or CSF NfL detected by ELISA		Detected by ELISA or SIMOA.

^a^ Applicable for possible prodromal MSA.

^b^ Division into motor subtypes is not applicable for possible prodromal MSA category.

^c^ Applicable for clinically established and clinically probable MSA.

Abbreviations: MSA, multiple system atrophy; MRI, magnetic resonance imaging; FDG‐PET, fluorodeoxyglucose‐positron emission tomography; MSA‐P, MSA‐parkinsonian type; MSA‐C, MSA‐cerebellar type; PSG, polysomnography; REM, rapid eye movement; OH, orthostatic hypotension; EMG, electromyography; MUP, motor unit potential; PSP, progressive supranuclear palsy; PD, Parkinson's disease; CSF, cerebrospinal fluid; PMCA, protein misfolding cyclic amplification; RT‐QuIC, real‐time quaking‐induced conversion; NfL, neurofilament light chain; ELISA, enzyme‐linked immunosorbent assay; SIMOA, single molecule array.

### Structural Brain MRI for Possible Prodromal MSA

Brain MRI markers of atrophy and diffusivity changes are applicable as biomarkers for the clinically established MSA criteria. Future studies are needed to establish a role for MRI in other categories.

### Brain fluorodeoxyglucose‐positron emission tomography (FDG‐PET)

Visual analysis of metabolic positron emission tomography (PET) with fluorodeoxyglucose (FDG) is potentially helpful to discriminate MSA from PD (for accuracy values of each affected brain region see Pellecchia et al^46^). Hypometabolism of the (posterior) putamen, pons, and cerebellum (which may be more pronounced in the striatum or in the pons and cerebellum, depending on the predominant motor presentation) is suggestive of MSA.^64^, ^65^, ^66^, ^67^ Cerebellar hypometabolism may also occur in other causes of cerebellar degeneration (eg, paraneoplastic or genetic ataxia).

### Cardiac ^123^I‐MIBG‐Scintigraphy for Clinically Established and Clinically Probable MSA

^123^I‐MIBG‐scintigraphy is a supportive diagnostic marker for clinically established and clinically probable MSA categories (abnormal findings are an exclusion criterion for possible prodromal MSA). Normal early (sensitivity: 83%, specificity: 89%) and delayed heart to mediastinum ratio (sensitivity: 90%–94%, specificity: 80%–83%) are typically observed in MSA, in contrast to decreased norepinephrine analogue uptake indicating myocardial postganglionic sympathetic dysfunction in PD.^68^, ^69^, ^70^ Reduced sympathetic innervation may occur due to medications affecting noradrenaline transporter and vesicular storage, structural heart disease (cardiomyopathy, atherosclerotic coronary artery disease), and other causes of small fiber neuropathy (diabetes mellitus). Mildly reduced ^123^I‐MIBG uptake in some MSA patients and normal uptake in early stages of PD further limit diagnostic accuracy.^71^, ^72^, ^73^, ^74^

### PSG‐Proven RBD for Clinically Established and Clinically Probable MSA

RBD is a common finding in both prodromal and established synucleinopathies, and is useful for the differentiation between MSA and non‐synucleinopathy disorders such as PSP and other causes of ataxia.^75^ Diagnosis of RBD requires repeated episodes of behavior or vocalization that are either documented by PSG to arise from REM or are presumed to arise from REM based on reports of dream enactment, and evidence of REM sleep without atonia on PSG. When REM sleep without atonia is not observed, the diagnosis may be given on a provisional basis when other clinical findings are strongly suggestive.^76^ As a supportive diagnostic marker it is applicable for clinically established and clinically probable MSA categories (PSG‐proven RBD is an essential feature of possible prodromal MSA).

### Supine Plasma Norepinephrine Levels

Plasma norepinephrine levels are relatively preserved in MSA (>100 pg/mL), but reduced in PD with nOH and in autoimmune or Lewy body isolated autonomic failure (for MSA vs. PD sensitivity: 36%, specificity: 100%, for MSA vs. isolated autonomic failure sensitivity: 82%–100%, specificity: 75%–100%). Supine norepinephrine >100 pg/mL appears to be the most sensitive and specific predictor of phenoconversion to MSA in patients with isolated autonomic failure.^59^, ^60^

### Urodynamic Testing

Urodynamic testing includes filling cystometry and pressure flow studies. In addition to detrusor overactivity, bladder neck incompetence, external sphincter denervation, detrusor underactivity, and detrusor sphincter dyssynergia are often observed in MSA. In contrast, detrusor sphincter dyssynergia is uncommon in PD and the PVR volume is generally low.^36^

### Pelvic Floor Neurophysiology

Concentric needle electromyography (EMG) of external anal sphincter demonstrating more than 20% of motor unit potentials (MUPs) having a duration >10 ms, or the average duration of MUPs >10 ms, analyzed manually to include late components, is suggestive of MSA in patients with parkinsonism if other common causes are excluded (see Table 4). An abnormal sphincter EMG may also be seen in other neurodegenerative diseases (such as PSP and long‐standing PD). A normal EMG, six or more years into the disease, would argue against the diagnosis of MSA.^77^

### α‐Synuclein Oligomers in CSF

Different conformational strains of α‐synuclein in MSA and LBD are reflected in the different reaction kinetics of protein misfolding cyclic amplification (PMCA). α‐Synuclein aggregation occurred later, but maximum thioflavin T (ThT) fluorescence was higher in LBD, allowing for separation from MSA at 2000 arbitrary units (AU) cut off (sensitivity: 100%, specificity: 93%).^78^, ^79^ Maximum ThT fluorescence and reaction kinetics typical of MSA were observed in all patients with isolated autonomic failure, who later phenoconverted to MSA.^80^ Limited evidence from immunohistochemistry studies suggests presence of α‐synuclein pathology in autonomic nerves in the skin biopsy samples from patients with PD but not from patients with MSA corresponding to different sites of autonomic involvement in these disorders. There is a need for future studies using seeding assays on skin biopsies to define the utility of skin biopsy in distinguishing MSA from PD.

### Neurofilament Light Chain in CSF and Plasma

Neurofilament light chain (NfL) level in CSF >1400 pg/mL detected by enzyme‐linked immunosorbent assay (ELISA) yielded 97% sensitivity and 90% specificity to distinguish MSA from LBD. Moverover, CSF NfL correlates with plasma NfL concentrations.^36^, ^78^ In patients who progressed from isolated autonomic failure to MSA, CSF NfL level was increased already at the stage of isolated autonomic failure, in contrast to the patients who did not phenoconvert.^80^

## Conclusions

Here we propose new MDS MSA diagnostic criteria that are designed for clinical practice, inclusion of patients in clinical trials, and research studies. The MDS MSA Criteria Revision Task Force aims to conduct a validation exercise on the MDS MSA criteria, including a prospective multicenter clinical study and a clinicopathological study. In particular, validation is needed for the research category of possible prodromal MSA and for supportive biomarkers for MSA diagnosis. We acknowledge that MDS MSA criteria will need revision, as data from ongoing and future studies become available. A systematic methodology to pick up emerging diagnostic innovations, the so‐called horizon scanning,^63^ is proposed to regularly update the criteria.

## Author Roles

1. Research Project: A. Conception, B. Organization, C. Execution; 2. Statistical Analysis: A. Design, B. Execution, C. Review and Critique; 3. Manuscript Preparation: A. Writing the First Draft, B. Review and Critique.

Gregor K. Wenning: 1A, 1B, 1C, 2C, 3B

Iva Stankovic: 1A, 1B, 1C, 2C, 3A

Luca Vignatelli : 2A, 2B, 3B

Alessandra Fanciulli : 1C, 3B

Giovanna Calandra‐Buonaura : 2A, 2B, 3B

Klaus Seppi: 1C, 3B

Jose‐Alberto Palma: 1C, 3B

Wassilios Meissner: 1C, 3B

Florian Krismer: 1C, 3B

Daniela Berg: 1C, 3B

Pietro Cortelli: 1C, 3B

Roy Freeman: 1C, 3B.

Glenda Halliday: 1C, 3B

Günter Höglinger 1C, 3B

Anthony Lang: 1C, 3B

Helen Ling: 1C, 3B

Irene Litvan: 1C, 3B

Phillip Low: 1C, 3B

Yasuo Miki: 1C, 3B

Jalesh Panicker: 1C, 3B

Maria Teresa Pellecchia: 1C, 3B

Niall Quinn: 1C, 3B

Ryuji Sakakibara: 1C, 3B

Maria Stamelou: 1C, 3B

Eduardo Tolosa: 1C, 3B

Shoji Tsuji: 1C, 3B

Tom Warner: 1C, 3B

Werner Poewe: 1A, 1B, 1C, 3B

Horacio Kaufmann: 1A, 1B, 1C, 3B

## Financial Disclosures of All Authors for the Preceding 12 Months

Gregor K. Wenning: consultancy and lecture fees from AbbVie, Affiris, AstraZeneca, Biogen, Lundbeck, Merz, Novartis, Ono, Teva, and Theravance; and research grants from the FWF Austrian Science Fund, the Austrian National Bank, the US MSA‐Coalition, Parkinson Fonds Austria, and International Parkinson and Movement Disorder Society, outside the submitted work.

Iva Stankovic: lecture fees from PharmaSwiss.

Luca Vignatelli: nothing to disclose.

Alessandra Fanciulli: royalties from Springer Nature Publishing Group and Thieme Verlag; speaker fees and honoraria from International Parkinson Disease and Movement Disorders Society, Austrian Neurology Society, Impact Medicom, Abbvie, and Theravance Biopharma; and research grants from the Stichting ParkinsonFond, US MSA Coalition, Dr Johannes Tuba Stiftung, and the Österreichischer Austausch Dienst, outside of the submitted work.

Giovanna Calandra‐Buonaura: personal fees for speaking from AbbVie, BIAL, and Zambon.

Klaus Seppi: personal fees from Teva, UCB, Lundbeck, AOP Orphan Pharmaceuticals AG, Roche, Grünenthal, Stada, Licher Pharma, Biogen, BIAL, and Abbvie; honoraria from the International Parkinson and Movement Disorders Society; research grants from FWF Austrian Science Fund, The Michael J. Fox Foundation, and AOP Orphan Pharmaceuticals AG, outside the submitted work.

Jose‐Alberto Palma: research funding from the National Institutes for Health (NIH), The Michael J. Fox Foundation, MSA Coalition, Familial Dysautonomia Foundation, Food and Drug Administration (FDA); advisory board member for Takeda, Astellas, and Dr. Reddy's Laboratories; managing editor of *Clinical Autonomic Research*; principal investigator in studies funded by Biohaven Pharmaceuticals, Theravance Biopharma, and Biogen; salary from Novartis.

Wassilios G. Meissner: personal fees for editorial activities with Elsevier; consultancy from Lundbeck, Biohaven, Roche, Alterity, Servier, and Inhibikase; and teaching honoraria from UCB.

Florian Krismer: reports receiving personal fees from Institut de Recherches Internationales Servier, Clarion Healthcare, and the Austrian Society of Neurology; grant support from the MSA Coalition, outside of the submitted work.

Daniela Berg: consultancies/advisory boards for Biogen, BIAL, UCB Pharma GmbH, Zambon; honoraria for talks/lectures from AbbVie, Bayser, Biogen, BIAL, UCB Pharma GmbH, Zambon, and Desitin; grants/research funding from Deutsche Forschungsgemeinschaft (DFG), German Parkinson's Disease Association (dPV), BMBF, Parkinson Fonds Deutschland gGmbH, UCB Pharma GmbH, EU, Novartis Pharma GmbH, Lundbeck, and the Damp Foundation.

Pietro Cortelli: nothing to disclose.

Roy Freeman: personal compensation and/or stock options for serving on scientific advisory boards of AlgoRx, Applied Therapeutics, Clexio, Collegium, Cutaneous NeuroDiagnostics, Glenmark, GW Pharma, Glaxo‐Smith Kline, Eli Lilly, Inhibikase, Lundbeck, Maxona, Novartis, NeuroBo, Regenacy, Vertex, and Worwag; personal compensation for his editorial activities (Editor) with *Autonomic Neuroscience – Basic and Clinical*; research support from the NIH (1R01NS10584401A1, R01HL111465‐01A1).

Glenda Halliday: stock ownership in medically‐related fields – Cochlear (2004 onwards) and NIB Holdings (2007 onwards); consultancies ‐ committee work for the National Health and Medical Research Council (NHMRC); employment ‐ University of Sydney; royalties ‐ Academic Press, Elsevier, and Oxford University Press; grants ‐ NHMRC (1,095,127; 1,132,524; 1,176,607; 1,191,407), NIH (12967627), The Michael J. Fox Foundation, Shake‐it‐up Australia, MSA Coalition, Motor Neurone Disease Research Institute of Australia (MNDRIA), University of Sydney, and Aligning Science Across Parkinson's.

Günter Höglinger: funded by the Deutsche Forschungsgemeinschaft (DFG, German Research Foundation) under Germany's Excellence Strategy within the framework of the Munich Cluster for Systems Neurology (EXC 2145 SyNergy ‐ ID 390857198) and within the Hannover Cluster RESIST (EXC 2155 ‐ ID 390874280), the German Federal Ministry of Education and Research (BMBF, 01KU1403A EpiPD; 01EK1605A HitTau; 01DH18025 TauTherapy), European Joint Programme on Rare Diseases (Improve‐PSP), Deutsche Forschungsgemeinschaft (DFG, HO2402/18–1 MSAomics), VolkswagenStiftung (Niedersächsisches Vorab), Petermax‐Müller Foundation (Etiology and Therapy of Synucleinopathies and Tauopathies), participated in industry‐sponsored research projects from Abbvie, Biogen, Biohaven, Novartis, Roche, Sanofi, and UCB. He serves as a consultant for Abbvie, Alzprotect, Asceneuron, Bial, Biogen, Biohaven, Kyowa Kirin, Lundbeck, Novartis, Retrotope, Roche, Sanofi, and UCB; received honoraria for scientific presentations from Abbvie, Bayer Vital, Bial, Biogen, Bristol Myers Squibb, Kyowa Kirin, Roche, Teva, UCB, and Zambon; holds a patent Höglinger GU, Höllerhage M, Rösler T. Treatment of Synucleinopathies. United States Patent No.: US 10,918,628 B2, date of patent: February 16, 2021; and received publication royalties from Academic Press, Kohlhammer, and Thieme.

Anthony Lang: served as an advisor for Abbvie, Acorda, AFFiRis, Biogen, Denali, Janssen, Lilly, Lundbeck, Maplight, Paladin, Retrophin, Roche, Sun Pharma, Sunovion, Theravance, and Corticobasal Degeneration Solutions; received honoraria from Sun Pharma, AbbVie, and Sunovion; received grants from Brain Canada, Canadian Institutes of Health Research, Corticobasal Degeneration Solutions, Edmond J. Safra Philanthropic Foundation, The Michael J. Fox Foundation, the Ontario Brain Institute, Parkinson Foundation, Parkinson Canada, and W. Garfield Weston Foundation; received publishing royalties from Elsevier, Saunders, Wiley‐Blackwell, Johns Hopkins Press, and Cambridge University Press.

Helen Ling: CDB Solutions research grant.

Irene Litvan: her research is supported by the NIH grants: 2R01AG038791‐06A, U01NS100610, U01NS80818, R25NS098999, U19 AG063911‐1, and 1R21NS114764‐01A1; The Michael J. Fox Foundation, Parkinson Foundation, Lewy Body Association, CurePSP, Roche, Abbvie, Biogen, Centogene, EIP‐Pharma, Biohaven Pharmaceuticals, Novartis, Brain Neurotherapy Bio, and United Biopharma SRL ‐ UCB. She was a member of the Scientific Advisory Board of Lundbeck and is a scientific advisor for Amydis and Rossy Center for Progressive Supranuclear Palsy University of Toronto. She receives her salary from the University of California San Diego and as Chief Editor of *Frontiers in Neurology*.

Phillip Low: research funding from federal grants (NIH; FDA) R01 NS092625, 1U01NS122419‐01A1, FD‐R‐07290, industry funds (Biohaven, Theravance), Sturm Foundation, and Mayo Funds.

Yasuo Miki: nothing to disclose.

Jalesh Panicker: undertook this work at University College London Hospitals NHS Foundation Trust (UCLH)/ University College London (UCL) Institute of Neurology and is supported in part by funding from the United Kingdom's Department of Health National Institute for Health Research (NIHR) Biomedical Research Centres funding scheme.

Maria Teresa Pellecchia: personal fees for consultancy and lectures from Zambon, Orion, and Theravance; principal investigator in studies funded by Theravance, Zambon, and Sanofi.

Niall Quinn: nothing to disclose.

Ryuji Sakakibara: nothing to disclose.

Maria Stamelou: honoraria for consultancy for Roche, UCB, Biogen, and ITF Hellas.

Eduardo Tolosa: received honoraria for consultancy from TEVA, Bial, Prevail Therapeutics, Boehringer Ingelheim, Roche, and BIOGEN and has received funding for research from the Spanish Network for Research on Neurodegenerative Disorders (CIBERNED) ‐ Instituto Carlos III (ISCIII) and The Michael J. Fox Foundation for Parkinson's Research (MJFF).

Shoji Tsuji: grant support from Nobelpharma Co., Ltd; and consultancies from Sanwakagaku Kenkyusho, iTRS, and Ono Pharmaceutical.

Tom Warner: employment ‐ UCL and UCLH; grants ‐ Medical Research Council, Reta Lila Weston Medical Trust, Corticobasal Degeneration Solutions Inc., UCLH BRC, Association of British Neurologists, and RoseTrees Trust; journal editor ‐ *Basal Ganglia*, *British Medical Bulletin*.

Werner Poewe: personal fees for consultancy and lectures from Alterity, AbbVie, Affiris, BIAL, Biogen, Britannia, Lilly, Lundbeck, Neuroderm, Neurocrine, Roche, Takeda, Teva, UCB, and Zambon; and grant support from The Michael J. Fox Foundation and the EU Horizon 2020 programme.

Horacio Kaufmann: Editor‐in‐Chief of *Clinical Autonomic Research*, published by Springer‐Nature, serves as principal investigator of a study sponsored by Biogen MA Inc. (TRACK MSA, S19‐01846); received consultancy fees from Lilly USA LLC, Biohaven Pharmaceuticals Inc., Takeda Pharmaceutical Company Ltd, Ono Pharma UK Ltd, Lundbeck LLC, and Theravance Biopharma US Inc. He receives research support from NIH‐National Institute of Neurological Disorders and Stroke (NINDS), Familial Dysautonomia Foundation, and The Michael J. Fox Foundation.

## Data Availability

Data sharing not applicable to this article as no datasets were generated or analysed during the current study

## Appendix

Movement Disorder Society (MDS) Multiple System Atrophy (MSA) Criteria Revision Task Force roles

Task Force chairs: Gregor K. Wenning, Horacio Kaufmann; Oversight Committee members: Gregor K. Wenning (chair), Glenda Halliday, Horacio Kaufmann, Werner Poewe, Niall Quinn; Task Force members: Anthony Lang, Daniela Berg, Pietro Cortelli, Roy Freeman, Phillip Low, Wassilios Meissner*, Jalesh Panicker*, Ryuji Sakakibara*, Klaus Seppi*, Shoji Tsuji*; Task Force expert consultants: Günter Höglinger, Irene Litvan, Maria Teresa Pellecchia*, Maria Stamelou, Eduardo Tolosa; Neuropathology Support Team: Janice Holton, Helen Ling, Yasuo Miki, Tom Warner; Executive Team: Iva Stankovic (leader), Giovanna Calandra Buonaura^#^, Alessandra Fanciulli, Florian Krismer, Alberto Palma, Luca Vignatelli^#^; Consensus Development Panel: Gregor K. Wenning, Horacio Kaufmann, Glenda Halliday, Werner Poewe, Niall Quinn, Günter Höglinger, Anthony Lang, Irene Litvan, Wassilios Meissner, Maria Teresa Pellecchia; Writing Committee: Gregor K. Wenning, Horacio Kaufmann, Glenda Halliday, Werner Poewe, Niall Quinn, Günter Höglinger, Anthony Lang, Irene Litvan, Wassilios Meissner, Maria Teresa Pellecchia, Klaus Seppi, Iva Stankovic, Giovanna Calandra Buonaura, Alessandra Fanciulli, Florian Krismer, Alberto Palma, Luca Vignatelli.

* Task Force members who contributed, together with the Executive Team, to drafting the answers to the questions related to clinical presentation and diagnostic tests for MSA (ie, literature review).

^#^Methodology Team responsible for devising the methodology of the Delphi rounds and Consensus Conference.
